# Integration of the first physician assistants into Israeli emergency departments – the physician assistants’ perspective

**DOI:** 10.1186/s13584-018-0275-3

**Published:** 2019-02-15

**Authors:** Rina Maoz-Breuer, Oren Berkowitz, Rachel Nissanholtz-Gannot

**Affiliations:** 1 Myers-JDC-Brookdale Institute, JDC Hill, P.O.B. 3886, 91037 Jerusalem, Israel; 2 Department of Health System Management, Ariel University, University Hill, 40700 Ariel, Israel

## Abstract

**Background:**

A new role of Physician Assistant (PA) was introduced into Emergency Departments (ED) in Israel in 2016, as part of a larger effort to improve the quality of service in the EDs. When the new role was introduced, there was a fair amount of uncertainty about whether it would succeed, in light of ambivalence on the part of many ED nurses, and lack of clarity among ED directors about the necessity of a PA role, and about the extent to which PAs would be allowed to take on professionally meaningful tasks.

The first class to train PAs was run by the Ministry Of Health between May 2016 and August 2017, with 34 PA trainees participating. 17 out of 24 EDs across Israel partook in the integration of the new PAs. This study assessed how this initial phase of integration is proceeding, from the perspective of the PA trainees themselves.

**Methods:**

New PA trainees were surveyed at the beginning and end of their training. Likert scale responses were collected (using a scale of 1 to 6). Respondents were asked about difficulties in their previous profession, their motives for choosing the PA profession and their expectations for the new position. The follow-up survey included additional questions about their clinical activities. Descriptive and correlational statistics were performed.

**Results:**

In the first survey, PA trainees reported that their main difficulties as paramedics were lack of options for professional advancement and burnout. New PA trainees had initially very high expectations for professional challenge, professional status upgrade, personal fulfillment, career prospects and an increase in wages (average mean score 5.7). In the follow-up survey there was a large drop in all of their ratings (average mean score 3.8).

In the second survey, PA trainees reported spending the majority of their time evaluating, diagnosing and managing patients as opposed to preforming clinical procedures, such as inserting an IV, administrating medicine or applying casts. Despite their decreased expectations, they still felt that they were intellectually stimulated (5.3 average), given high levels of responsibility (4.8 average), and making significant contributions to the healthcare team and patients (average score of 5.5). All of the above were correlated with overall satisfaction. The main difficulties they reported were related to limited authority and further career advancement.

**Conclusions:**

The new Israeli PA role has officially been launched in emergency medicine. The first group of PA trainees report a positive, productive integration, and overall satisfaction levels with their new career are high. However, the PA trainees reported having experienced some difficulties along the way, and there was a large decrease in their overall expectations from the new position during their first year on the job. Since the subject of limited authority was found to be a substantial difficulty for the new PA trainees, the Ministry of Health should explore this issue and create a uniform policy on it.

## Introduction

A new role of Physician Assistant (PA) was introduced into Emergency Departments (ED) in Israel in 2016. By adding PAs, Israel has joined a global trend of creating new healthcare roles and positions as a response to various medical workforce problems, including shortages in primary care, rural areas, and other settings [1]. Israel has also been dealing with similar problems such as a shortage of health professionals, especially in the periphery, and shortages in some specialties more than others (geriatrics, anesthesiology, intensive care, surgery, and pediatric subspecialties) [2–4]. These problems have forced the Israeli government to plan ahead and find creative ways to avoid or circumvent shortages. Measures that have been taken include opening a new medical school in the periphery, enlarging the size of existing medical schools, and encouraging Israeli professionals who moved abroad to return to Israel. Another response that was adopted in this context was introducing Physician Assistant and nurse practitioners to fill some of the clinical provider gaps in the healthcare workforce [3].

The decision to create a PA position in Israel drew on a longstanding idea that had been debated in the past but had not matured until recently. In 2013, the Israeli Ministry of Health (MOH) assembled a committee to discuss whether there is a need for this position. It concluded that the health system could benefit from the addition of PAs. Additionally, it was decided that the first PA positions would be created based upon an existing profession that would undergo special training to serve as a PA, rather than creating a new profession. The profession that was chosen as the most suitable to undergo the career re-training in the EDs was that of paramedics due to their set of skills and experience with emergency medicine. Another benefit to using paramedics would be helping a profession that has a high burnout rate and a very high turnover rate [5].

Simultaneously, the MOH led a multi-pronged initiative to improve the quality and level of services in Emergency Departments (EDs). The main challenges facing EDs were a large increase in the burden caused by the growing number of patients, together with a shortage in trained emergency medicine staff in general, and physicians in particular. This led to long waiting times, patient dissatisfaction [6, 7], stress, burnout of ED staff, and an overall inability to provide sufficiently professional and efficient treatment. The main initiatives undertaken included setting quality standards, adding employment positions on the emergency medicine staff (physicians, nurses, etc.), hiring medical administrators, introducing new technologies and advanced computing systems, and introducing incentives programs for EDs that meet the standards. As part of that process, the MOH decided to create the new role of PA, a position that would be added to the existing ED staff. In doing so, the EDs would essentially be the first department to pick up the baton of integrating the new position [8, 9].

The Israeli current model for PAs is still far from the well-established USA model. Average USA PA training lasts 27 months. In the USA, PA’s are certified by the National Commission on Certification of Physician Assistants, and are required to pass a recertification exam every few years. PAs practice medicine in all specialties and in every work setting, with close to half of them working in outpatient offices or clinics [10]. In contrast, in Israel the training was designed to last for 11 months, there is currently no independent national certifying organization, there is no recertification requirement, and the practice setting is limited to the ED. When the new role was introduced, there was a fair amount of uncertainty about whether it would succeed, in light of strong resistance from various elements of the emergency medicine scene in Israel. The first obstacle was a resolute objection to the new position by the nurse’s union, due to the threat that this position imposes on the nurses’ position and authority in the ED. This is still an ongoing issue that some EDs are dealing with, to different degrees. Another strong opponent of this initiative was the Israeli emergency medicine organization Magen David Adom, where most of the paramedics were previously employed.^1^ Other issues that arose were a lack of clarity among ED directors about the necessity of a PA role, and uncertainty about the extent to which PAs would be allowed to take on professionally meaningful tasks [11].

Nonetheless, The first PA training course was launched by the MOH in May 2016 and was based at the Sheba Medical Center. The students had to be either paramedics with a bachelor’s degree and a minimum of 5 years of working experience, or foreign medical school graduates who had not passed the Israeli medical board exams. Students were hired by a specific emergency department before beginning the course, each ED had an option to hire between one and three PA trainees. The MOH pilot program funded each participant’s salary during the yearlong training. During this year, training and practice were blended. Students spent 1 day per week in didactic education in the MOH training center and the other part in their employer’s hospital doing clinical work. Additionally, the students received formal teaching and training sessions in the ED. The main topics covered in the curriculum of the classroom part of the training program were related to ED clinical medicine, ED systems and administration, and common ED procedures [12]. The team designing the curriculum wanted to ensure that the PA trainees would have the training necessary to perform a wide range of typical ED procedures. With regard to the practical training in the field, this was left largely to the discretion of the individual ED directors. The program was interrupted by a wage dispute that was resolved after 4 months, but which nonetheless resulted in a later than anticipated graduation date of October 2017 in order to make up lost didactic time.

Since this was a pilot project for a very promising initiative regarding the Israeli health workforce, it was important to study the experience of the PA trainees integration into the EDs, and to examine how they perceived their new career. As these are the first PAs being incorporated into the workforce in Israel, information about their experiences could serve to improve future PA training and absorption in Israel. In addition, their experience and recommendations can potentially pull in (or, alternatively, drive away) future candidates from pursuing this career path, and be an obstacle to the evolution of the PA profession in Israel. Therefore, following the first class of PA trainees, by evaluating their perceptions and experiences, is crucial for future development of the PA profession Israel.

In order to do that, we first set out to examine the characteristics of the new PA trainees as well as their motivation in choosing to take part in this innovative program. More specifically, we sought to explore their perceptions at the beginning and end of their training, their expectations and concerns, and any difficulties in their jobs as paramedics. Finally, it was important to determine the clinical activities and the responsibilities that they were given in the EDs.

## Methods

New PA trainees were surveyed in person during the second week of training and again 12 months later. We developed a structured questionnaire comprised of questions on a unipolar 6-point Likert scale ranging from “not at all” to “a very large extent”, in addition to basic demographic information. In the first survey, respondents were asked about difficulties in their current profession, motives for choosing the new career, and expectations regarding their future position as PAs. The second survey contained the same self-assessment questions. In addition, in order to assess the PA trainees’ clinical activities, the second survey also included a list of clinical activities performed in the ED.

Participation in the survey was voluntary and the questionnaire was anonymous. An explanation of confidentiality and protection of personal information was provided in writing to all participants. This study was granted exempt status by the Brookdale Research Institute internal review board.

Statistical analyses were performed using SPSS (IBM Corp. Released 2016. IBM SPSS Statistics for Windows, Version 24.0. Armonk, NY: IBM Corp.). Descriptive statistics were performed with mean, standard deviation, range, and proportion, as appropriate. Results are reported without inferential statistics since this group represents the full population of current PA trainees in Israel. Spearman’s correlation was calculated in order to assess the strength and direction of the relationship between satisfaction with the choice to become a PA and other work conditions.

## Results

Thirty-four PA students enrolled in the course. Thirty-one were trained paramedics (94%) with an average of 13 years of experience (SD = 5.4) and the remaining three were foreign educated MDs. Almost all of the participants completed the questioner, thirty-three PA trainees (97%) responded to the first survey and thirty-two (94%) responded to the second survey (Table 1). All of the paramedics completed the course. Only three foreign educated physicians joined the PA course and none of them completed the course. Therefore, while we had responses from them for both rounds of the survey, these were not included in this analysis due to incomplete data and the inability to protect their confidentiality in a stratified analysis.

**Table 1:** Characteristics of PA trainees

Characteristics of PA trainees		*N* = 31
Demographics		
Average age (SD)		40.6 (5.5) range: 32–53
Born in Israel		83%
Men		94%
Jewish		94%
Qualifications		
Average years of experience as a paramedic (SD)		12.7 (5.4) range: 5–30
Highest degree obtained		
• BSc.		76%
• MSc.		24%
Barriers in previous profession as paramedics	Mean^a^	SD
No professional advancement	5.6	1
Burnout	4.6	1.5
Difficulty working shifts	3.4	1.8
Dissatisfaction with current profession	2.7	1.8

^a^ Ratings from 1 (not at all) to 6 (to a very large extent)

**Fig. 1:**
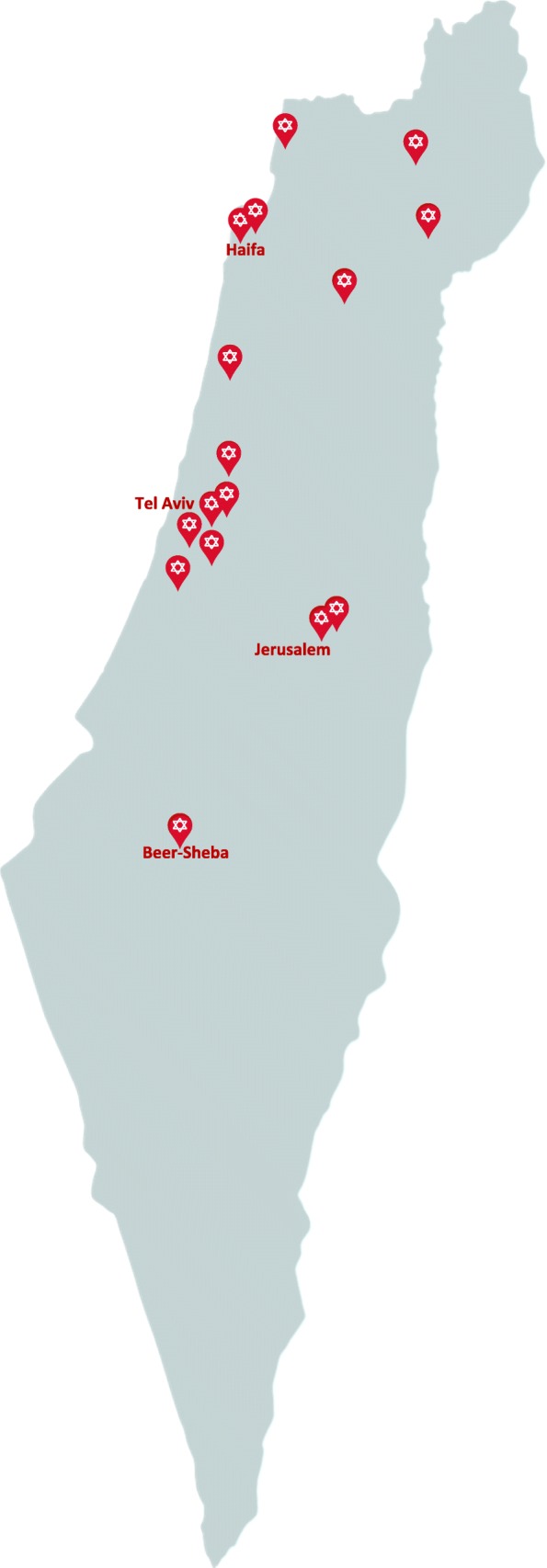
Hospitals with PA trainees in Israel

The PA trainees were incorporated into 17 out of 24 (71%) hospital EDs around Israel, with 41% of the EDs in the densely populated central regions. Outside of the central regions, 38% of the EDs were in the north, 15% in Jerusalem and 6% in the South (Fig. 1). On average, there were two PA trainees at each hospital. Participant average age was 41 years old (SD = 5.5). The vast majority where men (*n* = 30, 94%) and there were three women (6%). Of the participants, seven (24%) had a master’s degree.

### PA trainee clinical activities in the ED

**Fig. 2:**
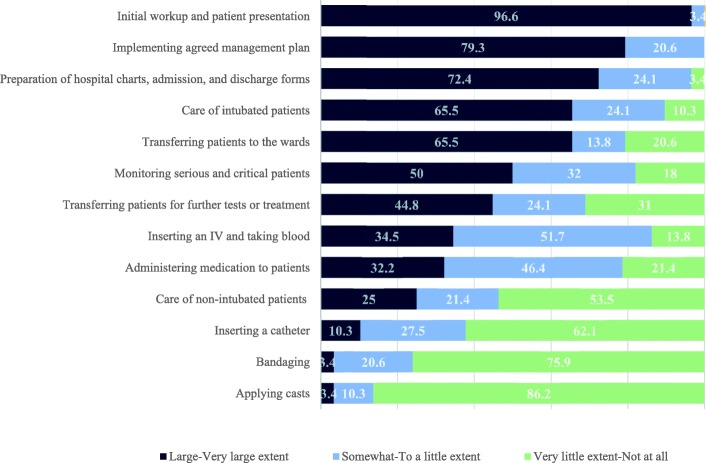
PA trainees clinical activities

There were several items that at least half of respondents agreed constituted a large or very large portion of their regular clinical activities. These activities were: evaluating new patients history, physical exam, and initial workup that they would present to the attending physician (97%), coordinating and implementing an agreed upon management plan (79%), preparing patient charts, admission papers, and discharge papers (72%), caring for intubated patients (66%), transferring patients to other units (66%), and monitoring patients in serious or critical condition (50%). PA trainees rarely inserted catheters or participated in wound care or casting fractures (Fig. 2).

### Career motivation factors at the beginning and at the end of training

Respondents were asked to rate the extent to which each of 4 factors constituted difficulties in their previous profession as paramedics, using a Likert scale of 1–6 with 1 representing no difficulty and 6 representing a great deal of difficulty. The respondents’ main difficulties in their previous paramedic profession were feelings of no options for professional advancement (average of 5.6, SD 1) and burnout (4.6, SD 1.5). Overall, they were not dissatisfied with their previous paramedic profession (2.7, SD 1.8) and had medium difficulty with working in shifts (3.4, SD 1.8) (Table 1).

Respondents were asked to rate the extent to which each of five motivational factors influenced their decision to become a PA (on a Likert scale of 1–6 with 1 representing no influence and 6 representing very high influence). In the first survey at the beginning of the course, respondents gave all five factors very high ratings of influence (average mean score 5.7) (Table 2), indicating that they were highly motivated to become PAs for the profession challenge, career prospects, and self-fulfillment. They rated their overall satisfaction with their decision to become a PA as very high at 5.8 (SD 0.5).

**Table 2:** Career motivation factors

	How relevant are each of these factors to you in your decision to become PA? (2016)		How relevant are each of these factors to you now? (2017)	
	Mean^a^	SD	Mean^a^	SD
Professional challenge	5.9	0.2	4.9	1.2
Personal fullfilment	5.8	0.5	4.4	1
Upgrading my professional status	5.8	0.5	4	1.4
Satisfaction with the decision to become PA	5.8	0.5	4	0.7
Career advancement	5.8	0.5	2.7	1.3
A chance of an increase in wages	5.3	0.9	3	1.1

^a^ Ratings from 1 (not at all) to 6 (to a very large extent)

At the end of their training, the PA trainees were again given these same items and asked to rate how much each factor resonated with them. All factor scores were rated lower at the end of their training (average mean score 3.8), but professional challenge (4.9, SD 1.2), professional status upgrade (4, SD 1.4), and personal fulfillment (4.4, SD 1) still resonated on the higher side of the scale on average. Respondents felt they had only a small chance of additional career advancement (2.7, SD 1.3) and increase in wages (3, SD 1.1). Overall satisfaction with the decision to become a PA remained positive (4, SD 0.7), albeit lower than at the beginning of the training.

### Working conditions and their correlation with satisfaction with the decision to become a PA

Respondents rated their level of agreement with a variety of statements about their work conditions, using a 6 point Likert scale. The statements with the highest level of agreement include three that related to the PAs’ positive contributions - to the patients (5.5, SD 0.6), the physicians (5.5, SD 0.7), and the ED (5.0, SD 0.7). Another statement with a high level of agreement was having exposure to a variety of interesting clinical cases (5.3, SD 0.8).

When Spearman’s correlation was calculated to look at the association between working conditions and satisfaction with the decision to become a PA, several moderate strength correlations were found. These were related to various aspects of the work: a sense of challenge and responsibility (rho = 0.47), the variety of clinical cases (rho = 0.44), and the use of professional skills (rho = 0.41). In addition, there was a moderate negative correlation between satisfaction and the perception that “the work is different from what we expected it to be” (rho = − 0.56) (Table 3).

**Table 3:** Work conditions and factors correlated with satisfaction

	Mean^a^	SD	Spearman rho [ƿ]
My contribution to the patients is significant	5.5	0.6	0.23
My contribution to the ED physicians is significant	5.5	0.7	0.13
I am exposed to a variety of interesting clinical cases at work	5.3	0.8	0.37
My contribution to the ED is significant	5	0.7	0.25
I believe there is a market demand for PAs	5	1.3	0.1
I believe I can build myself a position in the ED	4.9	1.1	0.37
I am happy to belong to the ED team	4.8	1	0.49
I have feelings of challenge and responsibility at work	4.8	1.2	0.47
I am exposed to a variety of interesting clinical cases at work, more than in my previous job	4.8	1.3	0.44
I have better hours compared to my previous work	4.8	1.8	0.41
I have practice variation	4.4	1.3	0.37
I feel that my position/status is not what I expected it to be	4.3	1.3	−0.56
My work allows me to use my professional skills	4.2	1	0.41
As a PA trainee, I do a lot of menial work	3.8	1	0.02
I have more treatment authority than in my previous job	2.2	1.3	0.29

^a^ Ratings from 1 (not at all) to 6 (to a very large extent)

### Perceived obstacles and challenges in the workplace

After a year of work in the EDs, PA trainees were asked to rate the extent to which they had encountered several potential obstacles and challenges in the workplace. The obstacles and challenges encountered most frequently, to a large or very large extent, were related to scope of practice (73%) and lack of further professional advancement (52%).

**Fig. 3:**
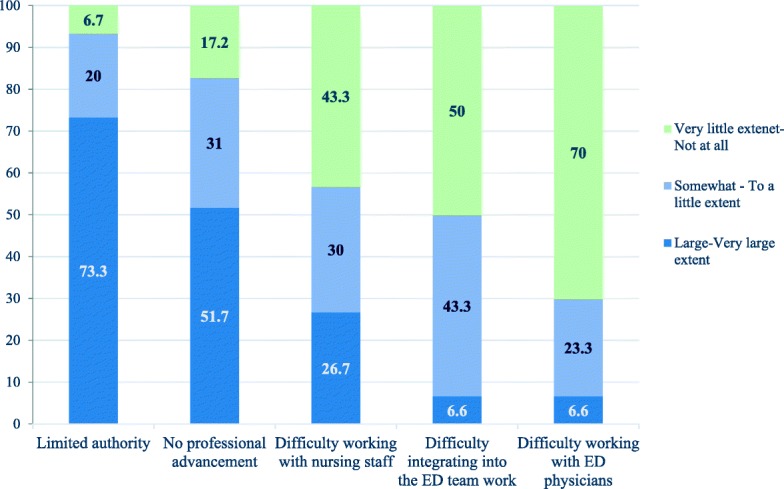
Perceptions of obstacles and work challenges

There was significant variation in the extent to which respondents reported resistance from the nursing staff. On the one hand, 27% reported feeling resistance when working with nursing staff to a large or very large extent; on the other hand, 43% reported very little or no resistance at all from the nursing staff. Relatively few respondents reported difficulties working with physicians or integrating with the ED team (Fig. 3).

## Discussion

The study objectives were to determine why people entered the PA profession, the PA trainees’ satisfaction and difficulties on the job, and the clinical activities of the new PA trainees. In the first survey, carried out during the second week of training, PA trainees reported that their main difficulties as paramedics were lack of options for professional advancement and burnout. New PA trainees had initially very high expectations for professional challenge, professional status upgrade, personal fulfillment, career prospects, and an increase in wages. In the follow-up survey a year later, there was a large drop in all of their ratings. The main difficulties they reported were related to limited authority and limited potential for further career advancement.

Our findings indicate that the position of PA trainees in the EDs is starting to form and take shape. The large amount of time that most PA trainees are spending evaluating, diagnosing, and managing patients demonstrates that they are being accepted and treated as an integral part of the clinical decision making team.

### Motivation and satisfaction over the course of the training program

We found that there was a change between the PA trainees’ initial levels of motivation and their feelings at the end of their training. Notably, despite this gap, their overall satisfaction levels with their career choice remained high. Our findings echo other studies worldwide regarding PAs reporting high levels of satisfaction with their job [13–15].

Among Israeli PA trainees, feelings about their new job were very likely tempered by the wage dispute that occurred; the dispute resulted in the PA trainees stepping out of classroom participation in protest for 4 months and then coming back to make up the lost time once it was resolved. This consequently led to a longer than anticipated training period and likely contributed to the decrease in satisfaction scores at the end of the training period, particularly with regards to salary and advancement prospects.

The high rating of all career motivational factors listed in the beginning, are consistent with workforce research on PAs in the USA. Studies found that PAs are largely motivated to choose a certain specialty based on feelings of self-worth, challenge, and intellectual fulfillment as well as an opportunity for higher wages and professional advancement [16–19].

### Perceived contributions and scope of authority in the transition from paramedic to PA

Respondents rated most of their working conditions positively. The highest scores were accorded to the PA trainees’ sense that they were making significant contributions to the healthcare team and to the patients. They felt challenged and felt that they received high levels of responsibility. The inverse item response helps validate this by showing lower levels of agreement when asked if they perform many menial tasks.

Interestingly, the lowest agreement score was related to a statement about having more treatment authority than in their previous profession and this response item appears as an outlier relative to the rest of the statements. Some of the direct patient care authority that the PA trainees had as paramedics have been reduced in the transition to the hospital workplace. For example, as paramedics, they were accustomed to delivering medication in the pre-hospital setting, whereas in the ED medication delivery was mostly restricted to nursing staff. Perhaps in their transition from very intimate one-on-one patient care in the ambulance to managing a panel of patients in the ED as clinical decision makers, they felt some degree of loss of involvement in bedside care.

There was a moderate degree of agreement with the statement that their new position/status is not what they expected it to be. This may reflect the degree of change from pre-hospital care to ED and might also be related to dissatisfaction with the salary dispute that occurred.

### The introduction of a new clinical position – Inter-professional and cultural considerations

The process of integrating a new clinical position into the health system is challenging to say the least. Along with the necessary regulatory changes, there has to be a significant culture change and buy-in from stakeholders for full integration and acceptance to occur. The USA has had 50 years of experience with the PA profession to cultivate the PA identity and create culture change. Several other countries that have implemented new PA professions in more recent years (such as Canada, England and The Netherlands) have also demonstrated acceptance and approval of the new PAs. This was shown through patient and provider surveys that paint a picture of PAs becoming important, integral, and respected parts of the clinical team despite the initial challenges [20–23].

Nursing and physician professional organizations in Israel held reservations and sometimes voiced strong oppositions to the creation of a PA role. Our survey results about perceived obstacles and challenges showed that close to half of the first group of PA trainees reported very little or no difficulty in working with nursing staff. Similarly, working with ED physicians and integrating into ED staff was in general, not perceived as an obstacle. These are positive signs overall, since integration and acceptance is the greatest barrier to overcome, whereas scope of practice and professional advancement can be changed through regulation once the PAs demonstrate their clinical competencies and the system is ready for it. This cohort of PA trainees with a paramedic background may also have benefited from an easier integration to the ED because of their familiarity with the environment from their previous work, especially working relationships with the nursing staff. Some nurses and PA trainees may have already known each other and worked together, as 87% of ED nurses encounter paramedics on a daily basis [5]. However, research that was done a few years ago showed that although ED nurses valued the paramedics’ clinical abilities, they found the idea of paramedics working as PAs less plausible than did physicians in that survey [24]. The authors have anecdotally heard that nursing leadership has reignited opposition to the PA role since the conclusion of this research project which may be linked to the nursing initiative to advance a nurse practitioner role. Although the early attitudes *vis a vis* working with nurses in this survey were positive, the long term relationship between nurses and PAs is still yet to be seen.

### Variation among hospitals

There was some variation among hospitals regarding which specific authorities were granted to the PA trainees. This is consistent with the differences of opinion voiced before the PA initiative was launched regarding the desired scope of practice- which authorities would be regulated by the MOH and which authorities would be delegated by the hospitals.

Variation in the utilization of PAs in the ED have also been found in previous studies in other countries, and it is common practice in places such as the USA for each hospital to determine its own PA scope of practice and to have its own credentialing agreements with its PAs, subject to adherence to governmental regulations [25].

Our results show that the subject of limited authority was a substantial difficulty for the new PA trainees. Accordingly, we propose that in Israel, which is a smaller and more highly regulated health system than the US system, a more uniform policy approach should be considered.

### Study strengths, limitations and directions for further research

One key advantage of this study is that it was planned ahead of the training program and conducted on the entire first class of PA trainees, following them prospectively as a cohort. Even though this first group is small, it provides a baseline for longitudinal comparison to conduct further studies in the future as the PA role evolves.

Our main research tool was a self-reporting survey, which carries an inherent risk of a non-differential measurement bias. The survey was designed with a broad range of questions, but there is the possibility that certain themes were missed and qualitative research may be necessary in order to explore additional questions related to PA integration in Israel. For example, we did not ask questions about advanced cardiac life support, resuscitation, or advanced airway management, which are things that we anecdotally heard the PA trainees perform quite well and frequently in the ED. This makes sense given their backgrounds as seasoned paramedics and should be included in any future study.

There were not enough foreign trained physicians who participated in this first group to report their data confidentially, but as more join in the future, there will be opportunity to report that group’s findings down the line. The regulation was also adjusted in 2017 to allow foreign educated PAs to join the Israeli PA course and we plan on studying their integration as a group, as well.

Additionally, future research in this area should investigate the stakeholders’ views of PA integration including physicians, healthcare managers, nursing staff, and patients in addition to measuring clinical quality and outcomes. The findings presented here are part of a larger research program that also examined physicians, ED directors and hospitals manager’s views of the integration. These will be reported in future publications.

## Conclusion

The new Israeli PA role has officially begun in emergency medicine. PA trainees report spending most of their time evaluating, diagnosing, and managing patients. The PA trainees, who were previously paramedics, chose to pursue this new role mostly for reasons of professional challenge, personal fulfillment, and career advancement. PA trainees feel intellectually stimulated, that they are making significant contributions to the healthcare team and to the patients and that they have been given high levels of responsibility, which are correlated with overall satisfaction.

Their main difficulties are related to scope of practice and further career advancement while integration into the clinical team is relatively less of a challenge. After a longer than anticipated training period, a wage dispute, and opposition from physician and nursing professional groups, the first group of PA trainees report a positive and productive integration and overall satisfaction levels with their new career are high. MOH should explore the issue of differences in PAs utilization and limited authority, and consider creating a uniform policy on PAs job description and authority in the ED.

## Abbreviations

ED: Emergency Departments
MOH: Ministry of Health
PA: Physician Assistant

## Footnotes

1 Magen David Adom was concerned that their employees, on whom they depend heavily, would leave them and join the PA program
